# Supplementary material for: Experimental evidence of seismic ruptures initiated by aseismic slip
Source: Nat Commun. 2024 Sep 19;15:8217. doi: 10.1038/s41467-024-52492-2 (PMC11410818; doi:10.1038/s41467-024-52492-2)
Supplement: Supplementary file 1 — Supplementary Information [file 41467_2024_52492_MOESM1_ESM.pdf]

Supplementary Information

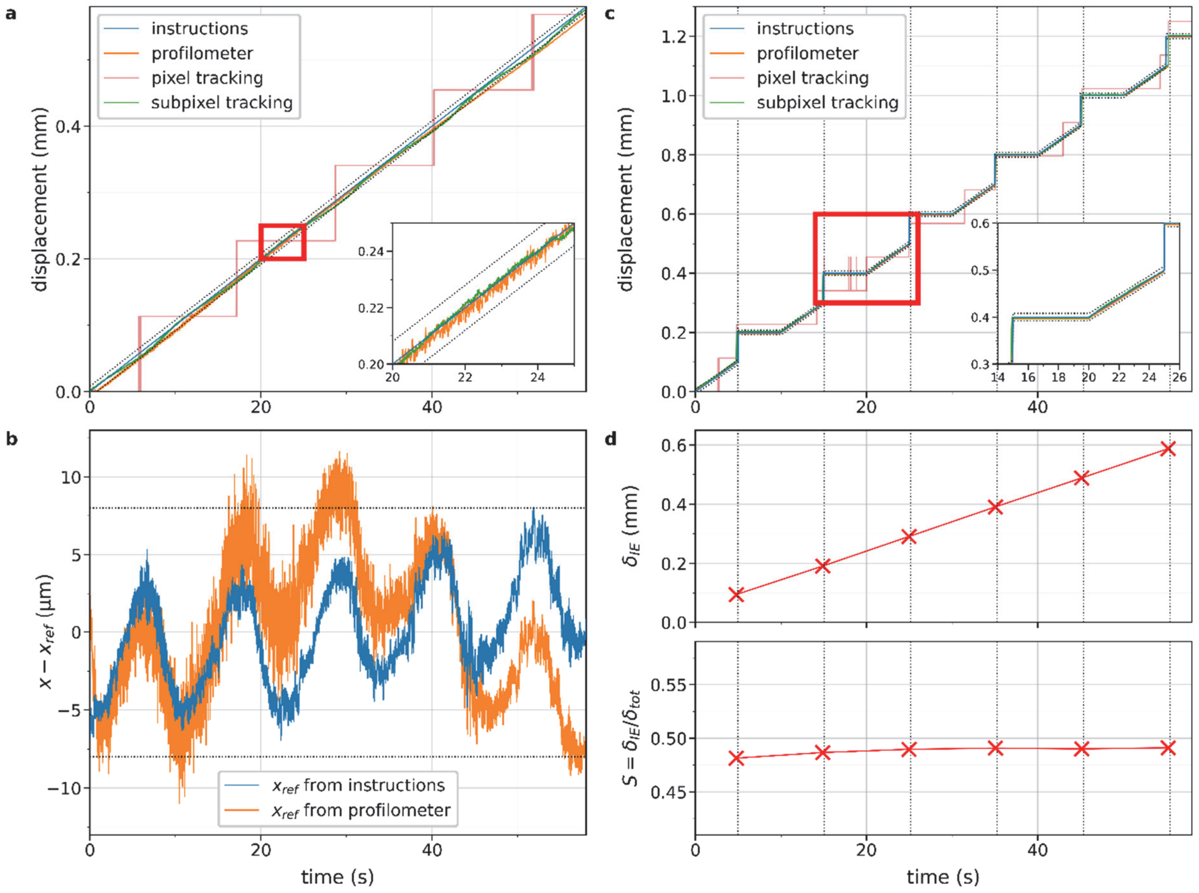

Fig. S1: Determination of the spatial resolution of the tracking method. (a) Displacement curves corresponding to a translation of the lower block (without shear resistance) at 10 μm/s imposed by the motorized stage. Instruction (blue line), block's displacement measured with an optical confocal profilometer (accuracy 0.4 μm, orange line), pixel resolution particle tracking (red line) and subpixel resolution particle tracking (green line) are superimposed. Black dotted lines indicate the ± 8 μm around the instruction curve corresponding to our subpixel tracking resolution. Inset: Zoom on the displacement curves (displacement from the pixel resolution tracking has been removed for clarity). (b) Difference between the displacement determined by the subpixel particle tracking measurement and the instruction (blue line) and the profilometer measurements (orange line). The standard deviation of $x - x_{ref}$ is $\sigma = 2.5$ µm, and $3\sigma \approx 8$ μm. Black dotted lines indicate the ± 8 μm resolution. (c) Displacement curves corresponding to a series of instructions emulating a stick-slip cycle with inter-event sliding, consisting of 6 repetitions of the following sequence: 100 μm at 20 μm/s, 100 μm at 10 mm/s (quasi instantaneous step), 5 s stops. Colored lines are as in (a). Inset: Zoom on one sequence (displacement from the pixel resolution tracking has been removed for clarity). (d) Temporal evolution of the cumulated inter-event slip without (top) and with (bottom) normalization. The non-normalized value is expected to be incremented by 100 μm after each inter-event period, and the normalized value is expected to be 0.5, since the same amount of slip (100 μm) occurs during a fast and a slow sliding event. The inter-event slip δ_{IE} is measured between 150 ms and 500 ms after the rapid event and 50 ms before the next rapid event (see Methods), which explains the difference between the expected (0.5) and measured values (0.495) for S.

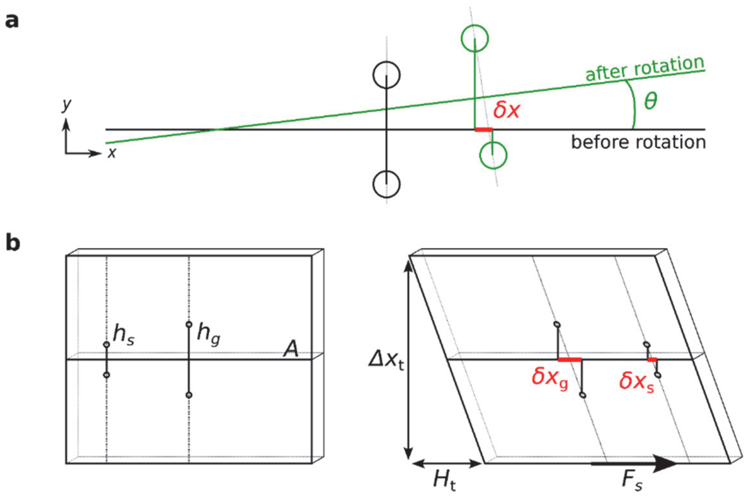

Fig. S2: Corrections applied to the tracking measurement. (a) Effect of the rotation of the interface relatively to the camera on the measured slip between the top and bottom grains. A rotation of an angle θ leads to a measured slip of δx. (b) Effect of the deformation of the blocks due to shear stress on the measured slip between the top and bottom grains. The measured slip goes as $\delta x = \frac{h\tau_{max}}{G}$, making a difference of measured slip between grains of different height.
